# The Ward’s Island “Difficulty” in New York

**Published:** 1890-06

**Authors:** 


### THE WARD'S ISLAND "DIFFICULTY" IN NEW YORK.

### Some Unpublished Correspondence.

53 West Forty-fifth Street,
New York, April 3d, 1890.

Dear Dr. Guernsey:—Owing to inability to attend a number of meetings of the Medical Board, I have not been well informed of the proceedings.

Recently I read in the March issue of *The New York Medical Times* the following resolution, passed by the Board January 2d, 1890:

"*Resolved,* that in the opinion of this Board, the only requirement as to belief and practice of a physician should be as follows: That in common with other existing associations which have for their object investigations and other labors which may contribute to the promotion of medical science, we hereby declare that we firmly believe the principle, *Similia similibus curantur,* to constitute the best general guide in the selection of remedies, and that we fully intend to carry out this principle to the best of our ability, yet this belief should not deter us from recognizing and making use of the results of any experience, and we shall exercise and defend the inviolable right of every educated physician to make use of any established principle in medical science, or any therapeutic fact founded on experiments and verified by experience, so far as in his individual judgment they shall tend to promote the welfare of those under his professional care."

The above resolution does not express my belief and practice, and as silence on my part would naturally be considered as indicating assent, I will now state my views, asking that they be read at the meeting to-night, and made a part of the record.

I believe that the words *Similia similibus curantur* formulate not "the best general guide," but a *law*—*a* GREAT NATURAL LAW to be applied as universally in the selection of drugs for the cure of sick people, as the law of gravity is in the realm of physics. Section 24 of Hahnemann's *Organon* sums the matter up as follows :

"There remains, accordingly, no other method of applying medicines profitably in diseases than the homœopathic, by means of which we select from all others that medicine (in order to direct it against the entire symptoms of the individual morbid case) whose manner of acting upon persons in health is known, and which has the power of producing an artificial malady the nearest in resemblance to the natural disease before our eyes."

I also believe that the incurably sick are best treated by *Similia*. They have thereby less suffering, longer life, and easier death.

My faith is founded upon large experience. Never has Homœopathy failed when subjected to the crucial test as directed by Hahnemann. For many years he and his followers have challenged disproof, but in vain. The challenge is yet open.

Believing the homœopathic to be the best way, I need not resort to any other. Any implication that an extremely sick person can be helped, somehow, by dropping the similar and taking up some other method, or by trying to combine methods, betrays a lack of faith in the law. Such patchwork is scouted by every true homœopath. The more desperate the case, the closer must we stick to the law, if we would cure.

My position is well understood at the Hospital and by the Medical Board. One of the original twenty-four members who received a sacred trust from the city authorities, I have steadfastly adhered to our mutual pledge made at our first meeting for organization, at the office of Dr. William H. White, to stick to the law. For my part, the promise was made *con amore.*

Nineteen years active practice of pure Homœopathy, teaching in college and hospital both didactic and clinical, and advice given in counsel with my professional brethren, all show a consistent record for *Similia*, to the best of my ability, to which all who know me will attest. The public have a right to expect of me a mode of practice which is consistent with my professions. If I ever cease to be a homœopath, I shall sever my connection with all homœopathic institutions and societies, and seek affiliation where I belong. The world is wide.

The resolution which called forth this letter was published in your journal. By right this should appear there also.

Very truly yours,

EDMUND CARLETON.

To Egbert Guernsey, M. D., President Medical Board Homœopathic Hospital, Ward's Island, New York.

---

To the foregoing letter Dr. Guernsey replied as follows:

NEW YORK, April 21st, 1890.

MY DEAR DOCTOR:—As you are probably aware, when the hospital on Ward's Island came into our hands Mr. Brennan insisted it should be called Homœopathic, and it was so called in spite of our protest. Notwithstanding it was to be under the control of our school, we insisted it should simply be designated as the Ward's Island Hospital. Have you any objection to our asking the Commissioners to change the name of the hospital to the one first proposed by the representatives of our school, viz.: THE WARD'S ISLAND HOSPITAL? Of course, there will be no change in treatment!

Very respectfully,

EGBERT GUERNSEY.

---

Dr. Carleton's rejoinder to the letter of Dr. Guernsey is herewith given:

NEW YORK, April 24th, 1890.

EGBERT GUERNSEY, M. D.

MY DEAR DOCTOR:—Your letter of the 21st instant received.

I have no recollection of the matter referred to. You have presented a subject which is entirely new to me. I supposed that we were to have a homœopathic hospital in fact and in name. To abandon homœopathic practice, wholly or partially, or to drop the distinctive title, at the present time, would be, to my mind, disastrous.

Very truly yours,
EDMUND CARLETON.

